# Dry Matter Yield of Maize (*Zea mays* L.) as an Indicator of Mineral Fertilizer Efficiency

**DOI:** 10.3390/plants10030535

**Published:** 2021-03-12

**Authors:** Piotr Szulc, Katarzyna Ambroży-Deręgowska, Hubert Waligóra, Iwona Mejza, Stanisław Grześ, Waldemar Zielewicz, Barbara Wróbel

**Affiliations:** 1 Department of Agronomy, Poznań University of Life Sciences, Dojazd 11, 60-632 Poznań, Poland; hubert.waligora@up.poznan.pl (H.W.); stanislaw.grzes@up.poznan.pl (S.G.); 2 Department of Mathematical and Statistical Methods, Poznań University of Life Sciences, Wojska Polskiego 28, 60-637 Poznań, Poland; Katarzyna.ambrozy-deregowska@up.poznan.pl (K.A.-D.); iwona.mejza@up.poznan.pl (I.M.); 3 Department of Grassland and Natural Landscape Sciences, Poznań University of Life Sciences, Dojazd 11, 60-632 Poznań, Poland; waldemar.zielewicz@up.poznan.pl; 4 Department of Grasslands, Institute of Technology and Life Sciences, Falenty, Al. Hrabska 3, 05-090 Raszyn, Poland; b.wrobel@itp.edu.pl

**Keywords:** maize, fertilizer depth, juvenile phase, dry matter, SPAD, partial factor productivity of fertilizer (PFPF)

## Abstract

This study presents the results of 3-year field trials, whose purpose was to assess the dynamics of dry matter accumulation by maize depending on the placement depth of a two-component (NP) mineral fertilizer in the soil layer, type of nitrogen fertilizer and date of its application. Weather conditions, mainly thermal in the early growing season, had a significant effect on maize responses to placement depth of phosphorus starting dose in the soil profile. In the initial stage of maize development, the temperature determined plant growth to a significantly higher extent than the sum of rainfall. The dry matter yield of ears and whole plants showed a clear reaction to starter phosphorus fertilization, but the effect of the depth of fertilizer placement varied over the years, indicating a depth of 5 cm and 10 cm as advisable and recommended for agricultural practice. The PFPF_N_ (partial factor productivity of fertilizer nitrogen) and PFPF_P_ (partial factor productivity of fertilizer phosphorus) indices confirmed the significant effect of fertilizer (NP) placement in the soil profile, indicating row fertilizer application (regardless of the depth) as recommended to improve the efficiency of maize fertilization. The SPAD (soil plant analysis development) leaf greenness index turned out to be a sensitive indicator of maize response to fertilizer (NP) placement depth in the soil profile.

## 1. Introduction

In the early spring period of almost every year, the observed growth inhibition of young maize plants and the occurrence of nutrient deficiency, especially N and P [1]. This was due to the limited nitrogen uptake at soil temperatures below 5 °C and phosphorus at temperatures below 12 °C [2]. These unfavorable phenomena could be counteracted by increasing the concentration of missing nutrients in the soil solution by localized fertilization, named starter fertilization, performed together with sowing seeds. Starter fertilization is defined as introducing a small amount of nutrient (fertilizer) near the seed placement [3]. The purpose of this component application method is to provide the necessary and readily available nutrients (mainly P) to freshly germinated seeds, which are very sensitive to its lack or unavailability [1,4]. Phosphorus, as a nutrient, is of key importance in the plant’s energy processes, and directly in synergy with zinc (Zn), it contributes to strong root growth and development [5]. A properly developed root system with a high mass and volume develops better in terms of depth and width in the soil profile and utilizes nutrient reserves available in the soil [6]. A well-developed juvenile maize plant is more tolerant to unfavorable thermal and humidity conditions [7]. Plants with a good phosphorus supply better tolerate water shortages, low temperatures and are less susceptible to pathogens [8]. Faster initial growth leads to uniform maize development, lower weed infestation and better moisture retention in the soil [9]. The author’s study showed that the starter sowing of ammonium phosphate, a two-component NP fertilizer, significantly increased maize grain yield compared to broadcast fertilization [10]. At the same time, this method of fertilization significantly improved the indices of N [6] and P application [10] in maize cultivation, as compared to broadcast fertilization, which applies mineral fertilizer to the soil surface. In the opinion of this author, placing NP fertilizer at a depth of 5 cm should be an indication for agricultural practice. The natural abundance of phosphorus in the soil determined the effectiveness of the row application of NP fertilizer. The lower it is, the greater the effectiveness of this fertilization method. Therefore, the question arises whether row (starter) fertilization will also have a beneficial effect on both the initial maize growth, expressed as dry matter accumulation and on dry matter yield of straw, ears and whole plants. Numerous studies have shown that the application of starter fertilizer below the depth of seed placement is the most advantageous method [2,7]. Such placement of mineral fertilizer in the soil profile results in the easy availability of the nutrient, while limiting the damage to germinating seeds [11]. In turn, Drazic et al. [7] used sporadically (pointwise) mineral fertilizer in their study as a starter fertilization only in the zone accessible by the root system. This method of fertilizer application significantly reduced the amount of the applied mineral fertilizer dose (economic and environmental aspect), while not reducing the size of maize grain yield. Therefore, the aim of the conducted field research was to determine the impact of different placement depths of a two-component (NP) mineral fertilizer in the soil profile on the (i) dynamics of dry matter accumulation in the early stages of maize growth, (ii) dry matter yield of straw, ears and whole plants, (iii) SPAD leaf greenness index in the critical stages of maize growth.

## 2. Materials and Methods

### 2.1. Experimental Field

**Figure 1:**
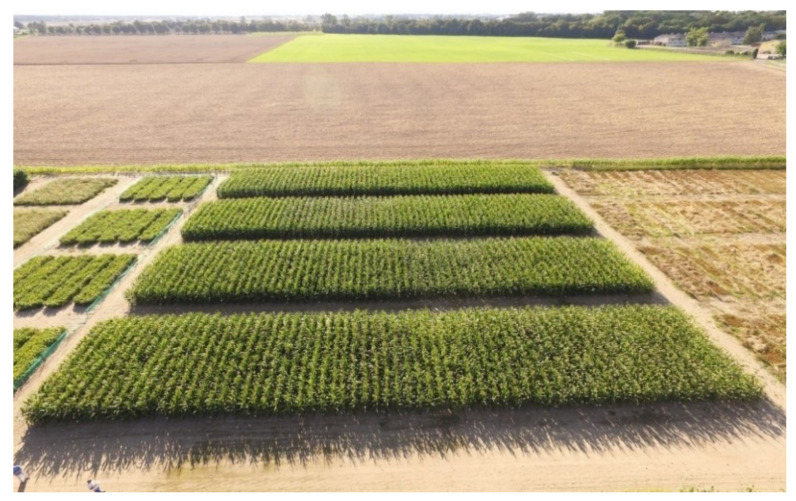
View of the maize experimental field in 2017. Photo taken from the drone (photo by the Szulc).

Field experiment was carried out at the Department of Agronomy of Poznań University of Life Sciences, on the fields of the Gorzyń Experimental and Educational Unit, in the years 2016–2018. It was conducted for four years in the same split-split-plot design with 3 factors and 4 field replicates (Figure 1). The following variables were tested: A: 1st order factor: NP fertilizer sowing depth (A1: 0 cm (broadcast), A2: 5 cm (in rows), A3: 10 cm (in rows), A4: 15 cm (in rows)); B: 2nd order factor: type of supplementary nitrogen fertilizer (B1: ammonium nitrate, B2: urea); C: 3rd order factor: date of supplementary nitrogen fertilization (C1: before sowing, C2: top dressing in the BBCH Bundesanstalt Bundessortenamt und CHemische Industrie 15/16 stage). The same level of mineral fertilization (100 kg of N ha^−1^, 70 kg of P_2_O_5_·ha^−1^ and 130 kg of K_2_O·ha^−1^) was applied in all experimental objects. Fertilization was balanced against P, which was applied at the whole required dose in the form of ammonium phosphate (18% N, 46% P_2_O_5_), according to the experimental design under the 1st order factor. K fertilization was performed before maize sowing in the form of potassium salt (60%). Fertilizer coulters (on objects with initial fertilization) were set 5 cm aside from the seeds. The application depth of NP fertilizer was according to the 1st order factor levels. Maize sowing was performed with a precision seeder, with a built-in granular fertilizer applicator (Monosem, Largeasse, France). The gross plot size was 24.5 m^2^ (length: 8.75 m, width: 2.8 m). The net plot area for harvesting was 12.25 m^2^. The row spacing was 0.75 m, while the kernel distance in a row was 0.18 m. The list of agriculture practices during the growing seasons of maize is listed in Table 1.

### 2.2. Meteorological Conditions

The thermal conditions during maize vegetation in the years of research were similar to each other and averaged 15.6 °C in 2016, 14.2 °C in 2017 and 16.6 °C in 2018. Definitely, greater differences between years occurred in total rainfall (Table 2). The highest sum was recorded in 2017 (553.0 mm), while the lowest sum of precipitation was recorded in the last year of the study: 230.3 mm. The calculated hydrothermal coefficients of water preservation according to Selyaninov (Table 2), considering comprehensively air temperature and atmospheric precipitation, allowed to one state that weather conditions for maize growth and development during two years of research were favorable (2016, 2017), while unfavorable in 2018 due to periodic soil moisture deficiencies. The sum of atmospheric rainfall in the period from maize sowing to the 8–9 leaf stage (BBCH 18/19) phase was as follows: 2016 (118.7 mm), 2017 (107.8 mm) and 2018 (46.4 mm).

The hydrothermal coefficient of water protection (K), according to Selyaninov, describes most comprehensively the influence of the thermal and humidity factor [12]:

$$K=\frac{10\;\times \;\mathrm{monthly}\;\mathrm{sum}\;\mathrm{of}\;\mathrm{rainfall}\;\mathrm{[mm]}}{\mathrm{number}\;\mathrm{of}\;\mathrm{days}\;\times \;\mathrm{average}\;\mathrm{daily}\;\mathrm{air}\;\mathrm{temperature}\;\mathrm{in}\;a\;\mathrm{month}\;({}^{\circ}C)}$$

The value of this coefficient for individual months in maize growing seasons is presented in Table 2.

Interpretation of the hydrothermal coefficient according to Selyaninov [12,13]:

- K > 1.5: excessive humidity for most plants,
- 1 < K < 1.5: humidity sufficient for most plants,
- 0.5 < K < 1.0: insufficient humidity for most plants,
- K < 0.5: drought.

### 2.3. Soil Conditions

The morphological structure of the experimental field was typical of the bottom moraine of the North Polish (Baltic) glaciation, the Poznań stadium. The parent materials of the soil were clay or sandy-loam formations. The terrain configuration showed little differentiation, and the dominant terrain was flat and low undulating. Typologically, the soils of the test field belonged to the black-earth type, a subtype of cambic black-earth, which belongs to the black-earth order. According to the international classification of WRB (World Reference Base), the studied soils should be classified as Phaeozemes, and according to the US Soil Taxonomy, as Mollisols. In terms of soil valuation, the experimental field was classified as IIIb class. The black-earth type includes soils where the direct influence of groundwater or heavy rainfall covers the lower and partly middle parts of the soil profile. In the surface horizons, rainfall and water management dominates, which can be modified to some extent by changing water properties of the deeper parts of the soil profile.

Soil abundance in basic macronutrients and pH is listed in Table 3. The magnesium content in the soil was determined by the Schachtschabel method, potassium by the Egner–Riehm method and phosphorus by the Olsen method. The content of nitrate nitrogen (N-NO_3_) and ammonium nitrogen (N-NH_4_) in the soil material was determined using the flow colorimetry method according to PN-R-04028:1997. The content of organic carbon in soil was determined by the Tiurin method.

### 2.4. Observations and Measurements

Representative samples of the fragmented plant material were placed in a standard cabinet dryer for large samples with a thermoregulator. The entire drying period was 20 h. The temperature in the dryer did not exceed 50 °C during the first six hours. The dryer was maintained at 105 °C during the remaining 14 h drying period. Temperature deviations during this period did not exceed 5 °C. After the 20 h drying period had elapsed, the plant material samples were maintained for two hours in the turned off but closed dryer. After cooling, the plant material samples were weighed.

#### 2.4.1. Determination of Dry Matter Accumulation Dynamics in the Initial Maize Vegetation Period

**a.** Single Plant Dry Weight in the 7–8-Leaf Stage (BBCH 17/18) and in the 8–9-Leaf Stage (BBCH 18/19)

Ten plants were collected from each plot for analysis. Samples were collected with a spade; subsequently, the root was separated from the aerial part of the plant. After drying, the dry matter of one plant was determined.

**b.** Dry Matter Determination in the 7–8-Leaf Stage (BBCH 17/18) and in the 8–9-Leaf Stage (BBCH 18/19)

The dry matter content of maize plants was determined knowing the fresh and dry matter of the harvested plants.

**c.** Dry Matter Yield Determination in the 7–8-Leaf Stage (BBCH 17/18) and in the 8–9-Leaf Stage (BBCH 18/19)

The yield of plant dry matter per unit area was calculated knowing the dry matter of a single plant and the quantitative status of plants in both studied maize development stages.

#### 2.4.2. Determination of Maize Dry Yield in the BBCH 63 Stage (The Beginning of Pollen)

During the maize harvest, weight measurements of whole plants were carried out, followed by ears alone; the total dry matter yield and yield structure were determined. The percentage of dry matter in the maize aerial parts was also determined in order to calculate dry matter yield of straw, ears and whole plants (straw + ear) per unit area.

#### 2.4.3. Estimation of Chlorophyll Content Expressed in SPAD Units

An optical device known in Europe as the Hydro N-Tester, and in the USA as SPAD-502, was used in the indirect method of determining the nutritional status of maize plants. This apparatus operates based on measuring the light absorption of the leaf at two wavelengths: 650 and 940 nm. The quotient of these differences is an indicator of chlorophyll content and is expressed in SPAD (soil and plant analysis development) units. A high coefficient of determination (R^2^) between apparatus readings and the amount of extracted chlorophyll was demonstrated depending on the species [14].

#### 2.4.4. Partial Factor Productivity of Fertilizer Nitrogen (N) and Phosphorus (P)

PFPF_N_ = P/Nr, (kg dm kg N);PFPFP = P/Pr, (kg dm kg P),

[15] where:
P—dry matter yield in an individual developmental phase of maize,Nr—nitrogen dose,Pr—phosphorus dose (pure component).


### 2.5. Statistical Analysis

Statistical analyses such as analysis of variance (ANOVA) and Tukey’s HSD (honestly significant difference) test for mean pair comparisons were performed in the study years separately and from 2016–2018, according to the experimental data models designed as a split-split-plot experiment [16,17]. All calculations were carried out using the Statistica 13 software package (2017) and MS Excel software. Statistical significance was defined at *p*-value ≤ 0.01 or *p*-value ≤ 0.05 depending on the source of variation.

## 3. Results

The different weather conditions in the study years 2016–2018 were reflected in all of the considered traits, i.e., the dry matter yield and leaf greenness index (SPAD index) at all tested plant growth dates, and partial factor productivity of fertilizer with P (PFPF_P_) and N (PFPF_N_).

### 3.1. Dry Matter Yields

Irrespective of the experimental factors (Table 4), significantly the highest dry matter yields were recorded in 2018 for both plant growth dates (274.61 kg dm ha^−1^ for BBCH 17/18 and 748.45 kg dm ha^−1^ for BBCH 18/19). In the remaining years of the study, the differences between the mean dm yields for BBCH 17/18 and for BBCH 18/19 were not significant.

Over the study years, the results also indicate the significant impact of NP fertilizer sowing depth (A) on the dry matter yield of maize. Irrespective of the year and other factors (Table 4), the use of a depth of at least 5 cm resulted in a significant increase in dry matter yields at both plant growth dates in compared to the fertilizer sowing depth A1: 0 cm (broadcast). The highest mean dry matter yields were obtained at BBCH 17/18 for a depth of 5 cm (198.64 kg dm ha^−1^) and at BBCH 18/19 for a depth of 5 cm (621,98 kg dm ha^−1^), but they did not differ significantly from the means for the other fertilizer sowing depths, 10 cm (in rows) and 15 cm (in rows).

No significant interaction was found between year of research and type of supplementary nitrogen fertilizer (B). Over the study years and other factors, at both plant growth dates, a significantly higher dry matter yield was obtained using urea as the supplementary nitrogen fertilizer than ammonium nitrate (Table 4).

For this trait, significant interaction was found between NP fertilizer sowing depth (A) and the year of research for BBCH 17/18 only. The results in Table 5 indicate that the greatest mean dry matter yields occurred in 2018 for both maize growth dates. However, for BBCH 17/18, dry matter yield increased significantly with the use of an NP fertilizer sowing depth (A) of at least 5 cm in rows. This effect was not significant for BBCH 18/19. In 2018, at both plant growth dates, the highest yield (although not significantly different compared with other depths) was obtained for a sowing depth of 10 cm (301.00 kg dm·ha^−1^ for BBCH 17/18 and 777.20 kg dm·ha^−1^ for BBCH 18/19).

A significant interaction was also obtained (Table 5) between the date of nitrogen application (C) and year (Y), but for BBCH 18/19 only. The highest dry matter yield was recorded in 2018 (Table 5). The date of supplementary nitrogen fertilization, before sowing or by top dressing at the BBCH 15/16 stage, was irrelevant, i.e., mean yields (761.27 kg dm ha^−1^ and 735.63 kg dm ha^−1^) did not differ significantly.

The results in Table 6 also indicate that variable climatic conditions in the years of the study significantly influenced the straw yield, ear yield and straw + ear yield of the maize variety used. The highest mean straw yields were obtained in 2017 (12,709.03 kg dm ha^−1^) and 2018 (11,317.28 kg dm ha^−1^). The difference between them is insignificant. In 2016, the straw mean yield was significantly lower. Moreover, for this trait there were no significant factors or interactions between factors, and no interactions with years.

In turn, the highest mean ear yields were obtained in 2017 (12,056.05 kg dm ha^−1^) and 2016 (11,493.24 kg dm ha^−1^). The difference between them is statistically insignificant. In 2018, the mean yield was significantly lower. The highest mean straw + ear yield was obtained in 2017 (24,765.09 kg dm ha^−1^), compared with 2016 and 2018, when the mean yields were similarly significantly lower.

The results in Table 6 indicate that only the mean ear yield and mean straw + ear yield increased significantly with the use of an NP fertilizer sowing depth (A) of at least 5 cm in rows in compared to the fertilizer sowing depth A1: 0 cm (broadcast). Other depths (10 and 15 cm) caused a negligible decrease in yields. The influence of factor A on the examined traits (the straw yield, ears yield and straw + ears yield) was independent of the year of the study (interactions with years are insignificant). Moreover, an insignificant influence of factor C on the straw yield, ears yield and straw + ears yield was noted.

### 3.2. Leaf Greenness Index (SPAD Index)

At the two stages of plant growth (BBCH 17/18 and BBCH 18/19) and during the flowering phase (BBCH 63), the leaf greenness index (SPAD) depends mainly on the NP fertilizer sowing depth (A) and year of research.

Regardless of the study years and other experimental factors, at stages BBCH 17/18 and BBCH 18/19, the highest mean values of the SPAD index were recorded for plants fertilized with nitrogen at a depth of 10 cm in rows, and statistically the lowest mean value when broadcast fertilization was used (Table 7). At BBCH 63, NP fertilizer sowing at a depth of at least 5 cm resulted in a significant increase in the leaf greenness index.

In turn, at BBCH 63 the highest SPAD index value was recorded for a 5 cm depth of fertilizer sowing, while at other depths (10 and 15 cm) the SPAD index values slightly decreased. They do not differ significantly from the mean SPAD index obtained for broadcast fertilization (Table 7).

Irrespective of the experimental factors, significantly the highest mean SPAD index values were recorded in 2017 for BBCH 17/18 (680.52), in 2017 for BBCH 18/19 (792.09), and in 2016–2017 for BBCH 63 (821.95, 817.06). The year 2018 was marked by a significant decline in the mean value of the SPAD index at all stages of plant growth (Table 7).

The results also indicate the significant impact of the date of supplementary nitrogen fertilization (C) on the plant’s SPAD index, both within years and irrespective of years, but only at BBCH 17/18. Regardless of the study year and other experimental factors, at stage BBCH 17/18, a significantly higher mean value of the SPAD index (591.17) was recorded for plants fertilized with supplementary nitrogen before sowing than for top dressing at the BBCH 15/16 stage (Table 7).

For this trait, significant interaction was found between the NP fertilizer sowing depth (A) and the year of research at stages BBCH 17/18 and BBCH 18/19 only.

**Figure 2:**
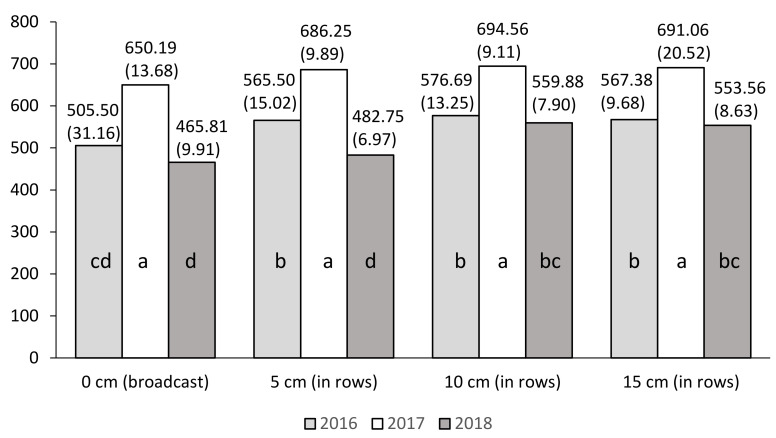
Mean values of index SPAD for the combinations of three years (Y) and four NP fertilizer sowing depths (A factor): BBCH 17/18. a, b, c, d: homogeneous groups (*p* ≤ 0.05). Standard errors are in brackets.

**Figure 3:**
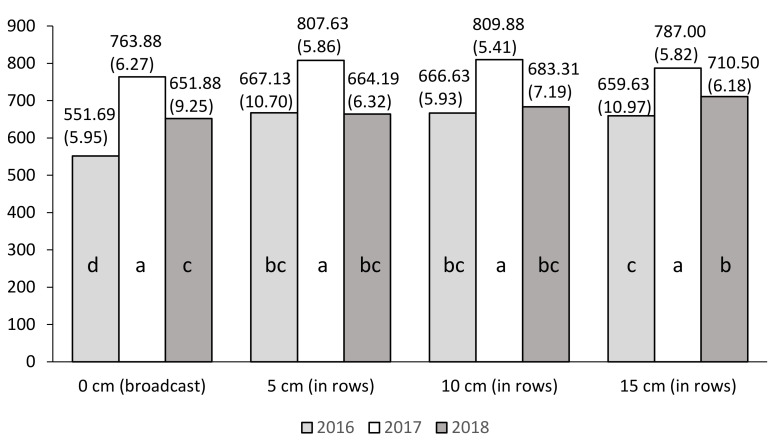
Mean values of index SPAD for the combinations of three years (Y) and four NP fertilizer sowing depths (A factor): BBCH 18/19. a, b, c, d: homogeneous groups (*p* ≤ 0.05). Standard errors are in brackets.

Figure 2 and Figure 3 indicate that significantly, the highest mean SPAD index values occurred in 2017 for both growth dates of maize. The mean values of the SPAD index in 2017 did not differ significantly on the use of an NP fertilizer sowing depth (A).

**Figure 4:**
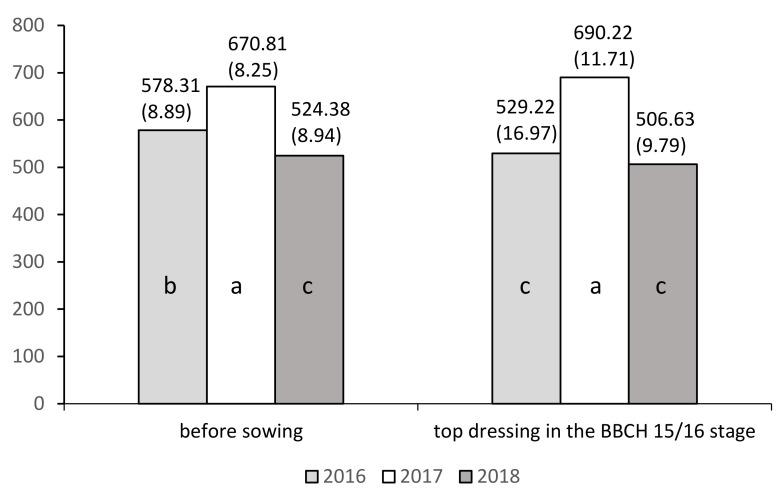
Mean values of index SPAD for the combinations of three years (Y) and two dates of nitrogen application (C factor): BBCH 17/18. a, b, c: homogeneous groups (*p* ≤ 0.05). Standard errors are in brackets.

For this trait, significant interaction was found between the date of supplementary nitrogen fertilization (C) and the year of research at stage BBCH 17/18. Figure 4 indicates that significantly the highest mean SPAD index (690.22) occurred in 2017 at BBCH 17/18 for both dates of additional fertilization application. The lowest mean values of the SPAD index were recorded in 2018 for both dates of additional fertilization application (524.38, 506.63), and in 2016 for top dressing (529.22).

### 3.3. Partial Factor Productivity of Fertilizer (PFPF)

In the study years, irrespective of the experimental factors, significantly the highest means of the partial factor productivity of phosphorus fertilizer (PFPF_P_) and the partial factor productivity of nitrogen fertilizer (PFPF_N_) with respect to dry matter yield of maize were recorded in 2018 for both plant growth dates (PFPF_P_: 8.92 kg dm·ha^−1^ P for BBCH 17/18 and 24.30 kg dm·ha^−1^ P for BBCH 18/19; and PFPF_N_: 2.75 kg dm·ha^−1^ N for BBCH 17/18 and 7.48 kg dm·ha^−1^ N for BBCH 18/19). In the remaining study years, the mean dry matter yields do not differ significantly among themselves, except for PFPF_N_ at BBCH 18/19, when significantly the lowest mean was achieved in 2017 (Table 8).

Over the study years, the results also suggest the significant impact of NP fertilizer sowing depth (A) on both PFPF_P_ and PFPF_N_. Irrespective of the year and other factors, the use of a depth of at least 5 cm resulted in a significant increase in dry matter yields at both plant growth dates. However, it should be noted that that increasing the sowing depth of NP fertilizer (10 and 15 cm) did not significantly differentiate the mean yields (Table 8).

Significant interaction was obtained between the NP fertilizer sowing depth (A) and year (Y) on both PFPF_P_ and PFPF_N_, but for BBCH 17/18 only. The highest means of dry matter yield were recorded in 2018 (Table 9).

No significant interaction was found between the year of research and type of supplementary nitrogen fertilizer (B) for either PFPF_P_ or PFPF_N_. Irrespective of the study year and other factors, at both plant growth dates, a significantly higher dry matter yield was obtained using urea as the supplementary nitrogen fertilizer than ammonium nitrate (Table 8).

Significant interaction also was obtained between the date of supplementary nitrogen application (C) and year (Y) for both PFPF_P_ and PFPF_N_, but for BBCH 18/19 only. The highest means of dry matter yield were recorded in 2018 (Table 9). The date of the application of supplementary nitrogen fertilization, before sowing or by top dressing at the BBCH 15/16 stage, was irrelevant: the mean yields did not differ significantly (Table 9).

The results in Table 10 and Table 11 suggest that variable climatic conditions in the study years significantly influenced the straw yield, ear yield and straw + ear yield of maize for both PFPF_P_ and PFPF_N_. Significantly the highest mean straw yield for PFPF_P_ was obtained in 2017 (412.63 kg dm·ha^−1^ P) and in 2018 (367.44 kg dm·ha^−1^ P). In 2016, the mean yield was significantly the lowest (Table 10). Significantly the highest mean straw yields for PFPF_N_ (127.09 and 113.17 kg dm·ha^−1^ N) were obtained from 2017–2018. In 2016, the mean yield was significantly lower (Table 11). Moreover, for this trait there were no significant factors or interactions between factors, and no interactions with years, for either PFPF.

The highest mean ear yields were obtained in 2016 and 2017 (the differences between them are insignificant) for both PFPF_P_ and PFPF_N_ (Table 10 and Table 11). In 2018, both mean yields were significantly lower. In turn, the highest mean straw + ear yield was obtained in 2017, compared with 2016 and 2018, when the yields were similarly significantly lower for both PFPF_P_ (Table 10) and PFPF_N_ (Table 11).

The results in Table 10 and Table 11 indicate that the mean ear yield and mean straw + ear yield increased significantly with the use of an NP fertilizer sowing depth (A) of at least 5 cm in rows. Other depths (10 and 15 cm) caused a negligible decrease in yields.

Moreover, an insignificant influence of factor C (date of supplementary nitrogen fertilization) on the straw yield, the ears yield and the straw + ears yield was noted for both PFPF_P_ and PFPF_N_.

## 4. Discussion

The obtained results indicate the significance of weather conditions that varied between study years on the size of plant dry matter yield in the 7–8- (BBCH 17/18) and 8–9-leaf stages (BBCH 18/19). On average, for the research years, the highest dry matter yield in the discussed developmental stages was recorded in 2018 (274.61 kg/ha and 748.45 kg/ha, respectively), while the lowest in 2017 (483.34 kg/ha) for the 8–9-leaf stage. As the water supply coefficient (K) increased, dry matter yield of maize at the BBCH 18/19 stage decreased. This arrangement of conditions was caused by the fact that precipitation in May was high (56.8 mm) in 2017, while air temperature was the lowest for that month, and amounted to 13.7 °C. On the other hand, for the lowest water supply coefficient in May 2018 (K = 0.33), dry matter yield in the initial period of maize growth was the highest. In the analyzed month, only 17.4 mm of rainfall was recorded, with an average air temperature of 16.9 °C (Table 2). Therefore, it can be concluded that it was the thermal factor that determined plant growth in the initial period of maize development, rather than the sum of precipitation. The results of the current study confirmed the previous literature reports on thermal requirements of maize [18]. Low soil and air temperature during sowing and in the early stages of maize growth is the main factor limiting its yield [19,20]. Considering the effect of the studied factors on the values of the discussed features, it was found that the depth of NP fertilizer sowing and the type of nitrogen fertilizer significantly influenced dry matter yield of maize plants. A significantly higher dry matter yield was recorded in maize fertilized in rows (irrespective of the depth) compared to broadcast fertilization. The result obtained in this study confirmed the earlier literature reports about the greater efficiency of maize fertilization using a localized method [21].

In the initial growth period, the poorly developed maize seminal root system can supply plants with minerals only if their concentration in the soil solution is sufficiently high [22]. On the other hand, the appropriate concentration of P in the soil solution is necessary to ensure the rapid growth of the maize root system and to reduce the effects of nutritional stress [23]. During this period, the concentration of P is very low compared to other anions, thus the proportion of phosphate ion in the sum of anions in the soil solution does not exceed a few percent. The effects of P deficiency in the juvenile maize growth period is therefore a classic example of the law of minimum. According to this rule, it is not possible to exploit the yield potential of a cultivated plant in the case of a deficiency of even a single mineral component, which was demonstrated in the present study. The only method to increase the availability of P in the initial period of maize growth is to place P in the immediate vicinity of the seeds using starter (localized) fertilization with this macroelement [7]. Considering the type of N fertilizer on the dynamics of initial maize growth, it was demonstrated that maize fertilized with urea was characterized by a significantly higher dry matter yield in the 7–8 and 8–9-leaf stage.

The application of nitrogen in the form of ammonium nitrate in the present study could result in the so-called physiological drought, leading to growth inhibition. Physiological drought is a period in which the plant cannot take up water from the environment, even though water is present there. The immediate cause is too high osmotic potential of the soil solution. The amount of water a plant takes up depends on the absorbing surface area and the difference in water potential. When the potential difference is too small, the plant does not take in enough water. The use of too much fertilizer causes hypertension of the soil solution and physiological drought. Borowiecki and Koter [24] obtained a different result concerning the influence of nitrogen fertilizer type on maize growth in the juvenile phase. The latter authors found that the emergence and growth of young maize was better in the presence of ammonium form of nitrogen or combined (ammonium and nitrate) than in the presence of only nitrate nitrogen or urea. This was probably due to the fact that ammonium nitrogen could be directly used for the biosynthesis of organic compounds (mainly proteins), which is especially important for the rapid growth of young plants, while nitrogen in the nitrate form requires a reduction in the plant organism and associated energy consumption. The poisoning of young maize seedlings may occur in objects fertilized with urea, which may be due to the harmful effect of ammonium nitrogen produced as a result of this compound hydrolysis. Niehues et al. [25] found that NH^4+^ applied on maize seeds (at doses >22 kg N ha^−1^), led to the damage of these seeds or seedlings, which resulted in reduced plant density and grain yield. According to Subedi and Ma [26], plant nitrogen malnutrition before the 8-leaf stage led to an irreversible reduction in the number of ears and potential kernels by up to 30%. According to [2], placing fertilizer in soil led to a higher nutrient concentration in various plant parts (by 3.7%) and nutrient contents in the aerial biomass (by 11.9%) compared to broadcast fertilization.

The yield of dry matter of ears and whole plants was also significantly dependent on the depth of fertilizer application. The obtained result in own research confirms previous research by Kruczek [27].

According to this author, row sowing was more effective compared to broadcast fertilization. Regardless of weather conditions and abundance of P in soil, row application of ammonium phosphate resulted in a significant increase in the dry matter yield of straw, ears and whole maize plants compared to broadcast fertilization. Chloroplast pigment contents also plays an important role in biosynthetic processes occurring in the green parts of the plant during the juvenile maize phase. Together with carotenoids, they absorb light energy, turning it into chemical energy, which is used in the process of organic compound synthesis from simple substances [28]. The higher their concentration, the higher the plant canopy productivity. The current study confirmed the above statement, because dry matter yield also increased with an increase in chlorophyll content expressed in SPAD units in the juvenile maize stages. Szulc and Waligóra [29] showed in a field experiment that the yield of maize grain also increased with an increase in chlorophyll content expressed in SPAD units at the 5–6-leaf stage. This proved that proper maize nutrition with N and P in the early developmental stage determined the final yield, which was confirmed by Subedi and Ma [26] and Gaj et al. [30].

N and P are one of the basic nutrients that are decisive in the intensification of plant production [31]. Current efforts to increase global cereal production, including maize, must be directed towards a more effective component utilization from a dose of mineral fertilizer. The increase in grain yield per N and P unit is particularly important due to concerns regarding the negative impact of an excess of these nutrients on the natural environment. This was also confirmed by Hassman et al. [32], who argued that the loss of nitrogen from arable fields could have a significant impact on environmental quality. A key role in this aspect may be played by greater ability of plants to uptake [33] and utilize a component [34] from the dose of mineral fertilizer. Hence, the constant direction of research on the role of nitrogen and phosphorus in shaping plant production is to determine biologically and economically justified optimal doses, taking into account factors affecting the uptake and utilization of these elements from mineral fertilizers. The present study showed that the partial factor productivity of fertilizer nitrogen (PFPF_N_) and phosphorus (PFPF_P_) indices were significantly determined by the sowing depth of NP fertilizer in the soil profile. A greater increase in dry matter yield per 1 kg of the applied nitrogen and phosphorus in the fertilizer was recorded for row sowing (regardless of the depth) compared to broadcast sowing. The distance of nitrogen fertilization placement from the plant root affects its uptake. As the distance of the fertilizer from the root system increases, there are fewer roots in a given soil volume, thus the probability that nitrogen will be absorbed by the roots decreases. Starter (row) fertilization increases the soil–fertilizer contact by placing nitrogen in the soil zone with a higher root concentration. This in turn increases the effectiveness of this method of sowing a nutrient [35,36].

## 5. Conclusions

Weather conditions, mainly thermal in the early growing season, had a significant impact on maize responses to placing depth of the P starting dose. The degree of maize reaction, expressed as the yield of plant dry matter in the BBCH 17/18 and BBCH 18/19 stages, increased under thermal stress conditions in the spring growing season. The dry matter yield of ears and whole plants showed a clear reaction to starter P fertilization, but the effect of the depth of fertilizer placement varied over the years, indicating a depth of 5 cm and 10 cm as advisable and recommended for agricultural practice.

The PFPF_N_ and PFPF_P_ indices confirmed the significant effect of NP fertilizer placement in the soil profile, indicating the row application of fertilizer (regardless of the depth) as recommended to improve the efficiency of maize fertilization. Urea fertilization at the BBCH 17/18 and BBCH 18/19 stages with a simultaneous placement of phosphorus in the soil significantly increased PFPF_N_ and PFPF_P_.

The SPAD leaf greenness index turned out to be a sensitive indicator of maize response to NP fertilizer placement depth in the soil profile. The date of supplementing nitrogen fertilizer (before sowing) significantly increased the value of this parameter only at the BBCH 17/18 stage.

## Figures and Tables

**Table 1:** Time points (dates) of agrotechnical treatments from 2016–2018.

Treatment Type	Years		
	2016	2017	2018
1. Deep plowing (30 cm) -treatment performer in the autumn the previous year	9.XI	26.X	20.XI
2. Harrow smoothing	1.IV	31.III	1.IV
3. Fertilizer sowing according to the experimental design	5.IV	20.IV	20.IV
4. Sowing fodder maize	28.IV cultivar P7905 (FAO 220)	25.IV cultivar P7905 (FAO 220)	24.IV cultivar P7905 (FAO 220)
5. Herbicide application ^1^-Maister Power (1.5 L ha^−1^) ^2^-Lumax 537.5 SE (3.5 L ha^−1^), ^3^-Lumax 537.5 SE (3.5 L ha^−1^),	28.V ^1^	28.IV ^2^	25.IV ^3^
6. Supplementary nitrogen fertilization	23.V	1.VI	14.V

**Table 2:** Average monthly air temperatures and monthly total precipitation for the growing season.

Years	Temperature (°C)							
	IV	V	VI	VII	VIII	IX	X	Average/Sum
2016	9.6	16.3	19.9	20.3	19	17.3	8.4	15.8
2017	7.3	13.7	17.4	18.0	18.9	13.3	10.6	14.2
2018	12.9	16.9	18.5	20.2	21.3	15.8	10.9	16.6
**Years**	**Rainfall (mm)**							
2016	47.3	47.3	123.8	132.8	50.3	4.6	105	511.1
2017	40.6	56.8	68.2	168.0	82.0	45.6	91.8	553.0
2018	36.2	17.4	25.6	70.5	11.6	44.2	24.8	230.3
**Years**	**Hydrothermal Coefficient of Water Protection (K) According to Selyaninov**							
2016	1.64	0.93	2.07	2.11	0.85	0.08	4.03	1.67
2017	1.85	1.33	1.30	3.01	1.39	1.14	2.79	1.82
2018	0.93	0.33	0.46	1.12	0.17	0.93	0.73	0.67

**Table 3:** Nutrient contents and soil pH before establishing the experiment in maize growing seasons.

Specification	Years			
		2016	2017	2018
P (mg P kg^−1^ dm of soil)		104.0	73.0	49.0
K (mg K kg^−1^ dm of soil)		97.0	108.0	116.0
Mg (mg Mg kg^−1^ dm of soil)		44.0	53.0	53.0
NH_4_NO_3_ (mg kg^−1^ dm of soil); 0.0–0.3 m		9.6	11.6	10.4
NH_4_NO_3_ (mg kg^−1^ dm of soil); 0.3–0.6 m		15.9	19.9	17.7
C, org. %		1.07	1.03	0.99
pH (1 mol dm^−3^ KCl)		4.6	5.6	5.1

dm—dry matter.

**Table 4:** Mean values of the traits for the years (Y), NP fertilizer sowing depth (A), type of supplementary nitrogen fertilizer (B), date of supplementary nitrogen fertilization (C).

Years and Factors	The Levels	BBCH 17/18 [kg dm ha^−1^]	BBCH 18/19 [kg dm ha^−1^]
Y	2016	134.21 ^b^ (3.52)	551.25 ^b^ (11.98)
	2017	149.02 ^b^ (3.22)	483.34 ^b^ (10.31)
	2018	274.61 ^a^ (5.54)	748.45 ^a^ (8.51)
A	0 cm (broadcast)	152.66 ^b^ (7.43)	534.09 ^b^ (20.13)
	5 cm (in rows)	198.64 ^a^ (10.37)	621.98 ^a^ (17.81)
	10 cm (in rows)	198.43 ^a^ (11.42)	616.23 ^a^ (20.36)
	15 cm (in rows)	194.04 ^a^ (10,41)	605.08 ^a^ (20.22)
B	ammonium nitrate	181.00 ^b^ (7.41)	580.15 ^b^ (14.54)
	urea	190.89 ^a^ (7.19)	608.55 ^a^ (13.89)
C	before sowing	185.79 ^a^ (7.26)	592.11 ^a^ (15.25)
	top dressing in the BBCH 15/16 stage	186.10 ^a^ (7.38)	596.58 ^a^ (13.27)

Values in columns marked with at least the same letter do not differ significantly at *p* ≤ 0.05. dm—dry matter. Standard errors are in brackets.

**Table 5:** Mean values for the combinations years (Y) × A factor and years (Y) × C factor.

Years (Y)	Depths of Fertilization (A)	BBCH 17/18 (kg dm ha^−1^)	BBCH 18/19 (kg dm ha^−1^)
2016	0 cm (broadcast)	115.22 ^d^ (6.03)	494.41 ^a^ (24.59)
	5 cm (in rows)	143.07 ^cd^ (5.43)	569.83 ^a^ (22.56)
	10 cm (in rows)	132.70 ^cd^ (8.20)	564.76 ^a^ (25.55)
	15 cm (in rows)	145.86 ^cd^ (6.03)	575.99 ^a^ (18.80)
2017	0 cm (broadcast)	124.43 ^cd^ (2.98)	417.46 ^a^ (19.09)
	5 cm (in rows)	163.98 ^c^ (7.73)	542.74 ^a^ (23.46)
	10 cm (in rows)	161.59 ^c^ (4.64)	506.74 ^a^ (13.63)
	15 cm (in rows)	146.07 ^cd^ (4.23)	466.41 ^a^ (11.86)
2018	0 cm (broadcast)	218.34 ^b^ (6.25)	690.40 ^a^ (15.42)
	5 cm (in rows)	288.89 ^a^ (9.63)	753.38 ^a^ (13.06)
	10 cm (in rows)	301.00 ^a^ (7.51)	777.20 ^a^ (18.79)
	15 cm (in rows)	290.20 ^a^ (6.33)	772.83 ^a^ (11.59)
**Years (Y)**	**Dates of Nitrogen Application** **(C)**	**BBCH 17/18** **(kg dm ha^−1^)**	**BBCH 18/19** **(kg dm ha^−1^)**
2016	before sowing	134.25 ^a^ (5.38)	548.37 ^b^ (16.47)
	top dressing in the BBCH 15/16 stage	134.17 ^a^ (4.62)	554.13 ^b^ (17.65)
2017	before sowing	145.79 ^a^ (4.12)	466.69 ^c^ (14.40)
	top dressing in the BBCH 15/16 stage	152.25 ^a^ (4.95)	499.98 ^c^ (14.37)
2018	before sowing	277.31 ^a^ (6.88)	761.27 ^a^ (12.88)
	top dressing in the BBCH 15/16 stage	271.90 ^a^ (8.77)	735.63 ^a^ (10.85)

Values in columns marked with at least the same letter do not differ significantly at *p* ≤ 0.05. dm—dry matter. Standard errors are in brackets.

**Table 6:** Mean values of the traits for the years (Y) and NP fertilizer sowing depth (A), type of supplementary nitrogen fertilizer (B), date of supplementary nitrogen fertilization (C).

Years and Factors	The Levels	Straw Yield (kg dm ha^−1^)	Ears Yield (kg dm ha^−1^)	Straw + Ears Yield (kg dm ha^−1^)
Y	2016	7090.90 ^b^ (177.92)	11,493.24 ^a^ (155.55)	18,599.77 ^b^ (249.24)
	2017	12,709.03 ^a^ (289.41)	12,056.05 ^a^ (191.35)	24,765.09 ^a^ (361.51)
	2018	11,317.28 ^a^ (232.46)	8932.31 ^b^ (271.57)	20,265.21 ^b^ (365.35)
A	0 cm (broadcast)	9877.40 ^a^ (458.04)	10,014.85 ^b^ (327.01)	19,933.92 ^b^ (552.90)
	5 cm (in rows)	10,645.63 ^a^ (441.62)	11,247.70 ^a^ (300.84)	21,893.33 ^a^ (554.15)
	10 cm (in rows)	10,552.21 ^a^ (416.04)	11,192.19 ^a^ (260.44)	21,744.41 ^a^ (478.86)
	15 cm (in rows)	10,414.38 ^a^ (450.67)	10,854.06 ^ab^ (334.44)	21,268.43 ^ab^ (516.26)
B	ammonium nitrate	10,431.53 ^a^ (284.62)	10,994.54 ^a^ (214.20)	21,436.48 ^a^ (348.43)
	urea	10,313.28 ^a^ (337.61)	10,659.86 ^a^ (227.88)	20,983.56 ^a^ (405.63)
C	before sowing	10,313.42 ^a^ (305.69)	10,991.61 ^a^ (212.54)	21,315.44 ^a^ (354.77)
	top dressing in the BBCH 15/16 stage	10,431.39 ^a^ (318.65)	10,662.79 ^a^ (229.47)	21,104.60 ^a^ (401.16)

Values in columns marked with at least the same letter do not differ significantly at *p* ≤ 0.05. dm—dry matter. Standard errors are in brackets.

**Table 7:** Mean values (SPAD) of the traits for the years (Y) and NP fertilizer sowing depth (A), type of supplementary nitrogen fertilizer (B), date of supplementary nitrogen fertilization (C).

Years and Factors	The Levels	BBCH 17/18	BBCH 18/19	BBCH 63
Y	2016	553.77 ^b^ (9.99)	636.27 ^b^ (7.49)	821.95 ^a^ (3.90)
	2017	680.52 ^a^ (7.21)	792.09 ^a^ (3.69)	817.06 ^a^ (5.57)
	2018	515.50 ^b^ (6.67)	677.47 ^b^ (4.54)	760.25 ^b^ (4.67)
A	0 cm (broadcast)	540.50 ^b^ (16.35)	655.81 ^b^ (13.30)	784.40 ^b^ (7.02)
	5 cm (in rows)	578.17 ^a^ (13.72)	712.98 ^a^ (10.74)	816.48 ^a^ (5.42)
	10 cm (in rows)	610.38 ^a^ (10.51)	719.94 ^a^ (9.87)	805.37 ^ab^ (6.57)
	15 cm (in rows)	604.00 ^a^ (12.00)	719.04 ^a^ (8.87)	792.78 ^ab^ (7.34)
B	ammonium nitrate	577.47 ^a^ (10.56)	699.86 ^a^ (8.16)	798.76 ^a^ (4.68)
	urea	589.05 ^a^ (8.90)	704.02 ^a^ (8.06)	800.75 ^a^ (4.97)
C	before sowing	591.17 ^a^ (7.95)	702.57 ^a^ (7.60)	800.00 ^a^ (4.73)
	top dressing in the BBCH 15/16 stage	575.35 ^b^ (11.27)	701.31 ^a^ (8.58)	799.51 ^a^ (4.72)

Values in columns marked with at least the same letter do not differ significantly at *p* ≤ 0.05. Standard errors are in brackets.

**Table 8:** Mean values of the traits for the years and (Y) NP fertilizer sowing depth (A), type of supplementary nitrogen fertilizer (B), date of supplementary nitrogen fertilization (C).

Years and Factors	The Levels	PFPF_P_ BBCH 17/18 (kg dm·kg^−1^ P)	PFPF_P_ BBCH 18/19 (kg dm·kg^−1^ P)	PFPF_N_ BBCH 17/18 (kg dm·kg^−1^ N)	PFPF_N_ BBCH 18/19 (kg dm·kg^−1^ N)
Y	2016	4.36 ^b^ (0.11)	17.90 ^b^ (0.39)	1.34 ^b^ (0.04)	5.51 ^b^ (0.12)
	2017	4.84 ^b^ (0.10)	15.69 ^b^ (0.33)	1.49 ^b^ (0.03)	4.83 ^c^ (0.10)
	2018	8.92 ^a^ (0.18)	24.30 ^a^ (0.28)	2.75 ^a^ (0.06)	7.48 ^a^ (0.09)
A	0 cm (broadcast)	4.96 ^b^ (0.24)	17.34 ^b^ (0.65)	1.53 ^b^ (0.07)	5.34 ^b^ (0.20)
	5 cm (in rows)	6.45 ^a^ (0.34)	20.19 ^a^ (0.58)	1.99 ^a^ (0.10)	6.22 ^a^ (0.18)
	10 cm (in rows)	6.44 ^a^ (0.37)	20.01 ^a^ (0.66)	1.98 ^a^ (0.11)	6.16 ^a^ (0.20)
	15 cm (in rows)	6.30 ^a^ (0.34)	19.65 ^a^ (0.66)	1.94 ^a^ (0.10)	6.05 ^a^ (0.20)
B	ammonium nitrate	5.88 ^b^ (0.24)	18.84 ^b^ (0.47)	1.81 ^b^ (0.07)	5.80 ^b^ (0.15)
	urea	6.20 ^a^ (0.23)	19.76 ^a^ (0.45)	1.91 ^a^ (0.07)	6.09 ^a^ (0.14)
C	before sowing	6.03 ^a^ (0.24)	19.22 ^a^ (0.49)	1.86 ^a^ (0.07)	5.92 ^a^ (0.15)
	top dressing in the BBCH 15/16 stage	6.04 ^a^ (0.24)	19.37 ^a^ (0.43)	1.86 ^a^ (0.07)	5.97 ^a^ (0.13)

Values in columns marked with at least the same letter do not differ significantlyat *p* ≤ 0.05. dm—dry matter. Standard errors are in brackets.

**Table 9:** Mean values for the combinations years (Y) × A factor and years (Y) × C factor.

Years (Y)	Depths of Fertilization (A)	PFPF_P_ BBCH 17/18 (kg dm·kg^−1^ P)	PFPF_P_ BBCH 18/19 (kg dm·kg^−1^ P)	PFPF_N_ BBCH 17/18 (kg dm·kg^−1^ N)	PFPF_N_ BBCH 18/19 (kg dm·kg^−1^ N)
2016	0 cm (broadcast)	3.74 ^d^ (0.196)	16.05 ^a^ (0.798)	1.15 ^d^ (0.060)	4.94 ^a^ (0.246)
	5 cm (in rows)	4.65 ^cd^ (0.176)	18.50 ^a^ (0.732)	1.43 ^cd^ (0.054)	5.70 ^a^ (0.226)
	10 cm (in rows)	4.31 ^cd^ (0.266)	18.34 ^a^ (0.829)	1.33 ^cd^ (0.082)	5.65 ^a^ (0.255)
	15 cm (in rows)	4.74 ^cd^ (0.196)	18.70 ^a^ (0.610)	1.46 ^cd^ (0.060)	5.76 ^a^ (0.188)
2017	0 cm (broadcast)	4.04 ^cd^ (0.097)	13.55 ^a^ (0.587)	1.24 ^cd^ (0.030)	4.17 ^a^ (0.181)
	5 cm (in rows)	5.32 ^c^ (0.251)	17.62 ^a^ (0.762)	1.64 ^c^ (0.077)	5.43 ^a^ (0.235)
	10 cm (in rows)	5.25 ^c^ (0.151)	16.45 ^a^ (0.442)	1.62 ^c^ (0.046)	5.07 ^a^ (0.136)
	15 cm (in rows)	4.74 ^cd^ (0.137)	15.14 ^a^ (0.385)	1.46 ^cd^ (0.042)	4.66 ^a^ (0.119)
2018	0 cm (broadcast)	7.09 ^b^ (0.203)	22.42 ^a^ (0.501)	2.18 ^b^ (0.063)	6.90 ^a^ (0.154)
	5 cm (in rows)	9.38 ^a^ (0.313)	24.46 ^a^ (0.424)	2.89 ^a^ (0.096)	7.53 ^a^ (0.131)
	10 cm (in rows)	9.77 ^a^ (0.244)	25.23^a^ (0.610)	3.01^a^ (0.075)	7.77^a^ (0.188)
	15 cm (in rows)	9.42 ^a^ (0.205)	25.09^a^ (0.376)	2.90^a^ (0.063)	7.73^a^ (0.116)
**Years (Y)**	**Dates of Nitrogen Application** **(C)**	**PFPF_P_** **BBCH 17/18** **(kg dm·kg^−1^ P)**	**PFPF_P_** **BBCH 18/19** **(kg dm·kg^−1^ P)**	**PFPF_N_** **BBCH 17/18** **(kg dm·kg^−1^ N)**	**PFPF_N_** **BBCH 18/19** **(kg dm·kg^−1^ N)**
2016	before sowing	4.36 ^a^ (0.175)	17.80 ^b^ (0.535)	1.34 ^a^ (0.054)	5.48 ^b^ (0.165)
	top dressing in the BBCH 15/16 stage	4.36 ^a^ (0.150)	17.99 ^b^ (0.573)	1.34 ^a^ (0.046)	5.54 ^b^ (0.177)
2017	before sowing	4.73 ^a^ (0.134)	15.15 ^c^ (0.468)	1.46 ^a^ (0.041)	4.67 ^c^ (0.144)
	top dressing in the BBCH 15/16 stage	4.94 ^a^ (0.161)	16.23 ^c^ (0.466)	1.52 ^a^ (0.050)	5.00 ^c^ (0.144)
2018	before sowing	9.00 ^a^ (0.223)	24.72 ^a^ (0.418)	2.77 ^a^ (0.069)	7.61 ^a^ (0.129)
	top dressing in the BBCH 15/16 stage	8.83 ^a^ (0.285)	23.88 ^a^ (0.352)	2.72 ^a^ (0.088)	7.36 ^a^ (0.109)

Values in columns marked with at least the same letter do not differ significantly at *p* ≤ 0.05. dm—dry matter. Standard errors are in brackets.

**Table 10:** Mean values of the traits for the years (Y) and NP fertilizer sowing depth (A), type of supplementary nitrogen fertilizer (B), date of supplementary nitrogen fertilization (C).

Years and Factors	The Levels	PFPF_P_ Straw Yield (kg dm·kg^−1^ P)	PFPF_P_ Ears Yield (kg dm·kg^−1^ P)	PFPF_P_ Straw + Ears Yield (kg dm·kg^−1^ P)
Y	2016	230.22 ^b^ (5.78)	373.16 ^ab^ (5.05)	603.89 ^b^ (8.09)
	2017	412.63 ^a^ (9.40)	391.43 ^a^ (6.21)	804.06 ^a^ (11.74)
	2018	367.44 ^a^ (7.55)	290.01 ^b^ (8.82)	657.96 ^b^ (11.86)
A	0 cm (broadcast)	320.69 ^a^ (14.87)	325.16 ^b^ (10.62)	647.21 ^b^ (17.95)
	5 cm (in rows)	345.64 ^a^ (14.34)	365.19 ^a^ (9.77)	710.82 ^a^ (17.99)
	10 cm (in rows)	342.60 ^a^ (13.51)	363.38 ^ab^ (8.46)	705.99 ^ab^ (15.58)
	15 cm (in rows)	338.13 ^a^ (14.63)	352.40 ^ab^ (10.86)	690.53 ^ab^ (16.76)
B	ammonium nitrate	338.69 ^a^ (9.24)	356.97 ^a^ (6.45)	695.99 ^a^ (11.31)
	urea	334.85 ^a^ (10.96)	346.10 ^a^ (7.40)	681.28 ^a^ (13.17)
C	before sowing	334.85 ^a^ (9.93)	356.87 ^a^ (6.90)	692.06 ^a^ (11.52)
	top dressing in the BBCH 15/16 stage	338.68 ^a^ (10.35)	346.19 ^a^ (7.45)	685.21 ^a^ (13.02)

Values in columns marked with at least the same letter do not differ significantly at *p* ≤ 0.05. dm—dry matter. Standard errors are in brackets.

**Table 11:** Mean values of the traits for the years (Y) and NP fertilizer sowing depth (A), type of supplementary nitrogen fertilizer (B), date of supplementary nitrogen fertilization (C).

Years and Factors	The Levels	PFPF_N_ Straw Yield (kg dm·kg^−1^ N)	PFPF_N_ Ears Yield (kg dm·kg^−1^ N)	PFPF_N_ Straw + Ears Yield (kg dm·kg^−1^ N)
Y	2016	70.91 ^b^ (1.78)	114.93 ^ab^ (1.56)	186.00 ^b^ (2.49)
	2017	127.09 ^a^ (2.89)	120.56 ^a^ (1.91)	247.65 ^a^ (3.62)
	2018	113.17 ^a^ (2.32)	89.32 ^b^ (2.72)	202.65 ^b^ (3.65)
A	0 cm (broadcast)	98.77 ^a^ (4.58)	100.15 ^b^ (3.27)	199.34 ^b^ (5.53)
	5 cm (in rows)	106.46 ^a^ (4.42)	112.48 ^a^ (3.01)	218.93 ^a^ (5.54)
	10 cm (in rows)	105.52 ^a^ (4.16)	111.92 ^ab^ (2.60)	217.44 ^ab^ (4.80)
	15 cm (in rows)	104.14 ^a^ (4.51)	108.54 ^ab^ (3.34)	212.68 ^ab^ (5.16)
B	ammonium nitrate	104.32 ^a^ (2.85)	109.95 ^a^ (2.14)	214.36 ^a^ (3.48)
	urea	103.13 ^a^ (3.38)	106.60 ^a^ (2.28)	209.84 ^a^ (4.06)
C	before sowing	103.13 ^a^ (3.06)	109.92 ^a^ (2.13)	213.15 ^a^ (3.55)
	top dressing in the BBCH 15/16 stage	104.31 ^a^ (3.19)	106.63 ^a^ (2.29)	211.05 ^a^ (4.01)

Values in columns marked with at least the same letter do not differ significantly at *p* ≤ 0.05. dm—dry matter. Standard errors are in brackets.

## Data Availability

The data presented in this study are available in this article.
